# Bioinspired super-tough polyurethane elastomers with block modules using sacrificial bonds

**DOI:** 10.1039/d5ra08303f

**Published:** 2026-01-16

**Authors:** Jian Li, Fubo Ma, Jintao Ji, Yuanzhi Qu, Xiaoxiao Ni

**Affiliations:** a State Key Laboratory of Deep Oil and Gas, China University of Petroleum (East China) Qingdao 266580 China cuplijian@sina.com; b School of Petroleum Engineering, China University of Petroleum (East China) Qingdao 266580 China; c Hebei Drilling and Production Equipment Manufacturing Branch, PetroChina Company Limited Renqiu 062552 China; d Drilling Fluid Research Institute, CNPC Engineering Technology R&D Company Limited Beijing 102206 China

## Abstract

Stretchable and self-healable elastomers with excellent mechanical properties can find attractive applications in electronic skin, soft robotics, and electrical devices. To date, it remains a huge challenge to synthesize self-healing elastomers that integrate extreme stretchability, relatively high toughness, and high self-recoverability. Herein, inspired by biological tissues and mussel byssus, we circumvent this dilemma by introducing multiple hydrogen bonds (UPy) and metal coordination bonds (DAP-Fe(iii)) into a linear polyurethane network. The self-complementary quadruple hydrogen-bond interactions between UPy dimers were incorporated as physical cross-linkages, with greatly enhanced mechanical strength and high stretchability. In addition, strong Fe-coordination bonds can readily break and re-form, a feature that facilitates energy dissipation during stretching, leading to significantly improved robustness while maintaining stretchability. The polyurethane elastomer exhibited all the desired properties, including high tensile stress (∼30 MPa), high stretchability (∼4100%), exceptional toughness (∼470 MJ m^−3^), excellent self-recoverability, and self-healing ability. This biomimetic strategy of using synergistic dynamic bonds as block modules is an alternative approach for obtaining advanced polymers.

## Abstract 2

A bioinspired polyurethane elastomer with multiple hydrogen bonds and metal coordination bonds was prepared, exhibiting desirable properties such as high tensile stress, stretchability, and exceptional toughness.

## Graphical abstract

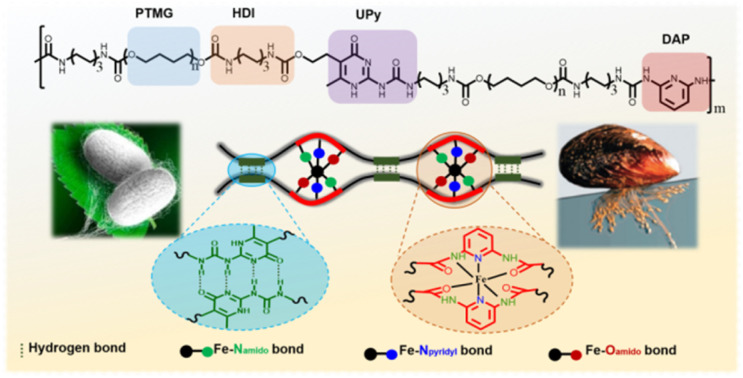

## 1 Introduction

Elastomers, with “soft-and-deformable” nature, are acknowledged as important functional materials due to their fascinating properties (high extensibility and recoverability) and similarities to biological tissues.^1–8^ In recent years, elastomers have been widely employed as scaffolds for smart, flexible, wearable, and stretchable devices.^9–13^ However, most elastomers suffer from poor mechanical properties, which severely hinder their practical applications—especially in load-bearing soft tissues requiring stiffness, toughness, fatigue resistance, and self-healing properties. To address this issue, considerable progress has been made in efforts to prepare robust, durable, and self-healable elastomers in recent years.^14–19^ Unfortunately, it remains challenging to obtain the desired elastomers that possess the properties of biological muscles—strong, elastic, self-healing, and self-recoverable.

As is well known, nature has evolved an intricate and intriguing strategy by developing complex hierarchical structures in biomaterials (such as mussel byssus^20,21^ spider silk^22^ and bone^23^) that can exhibit an unparalleled combination of stiffness and toughness. Inspired by nature, many smart methodologies—including various nanofillers,^24,25^ hierarchical “brick and-mortar” structure,^26,27^ microphase-separated system,^28,29^ double network system,^30,31^ and dynamic reversible reactions,^32,33^ have been explored into man-made polymers with the goal of achieving biomimetic strength and toughness. Due to their reversible properties, dynamic noncovalent interactions can efficiently dissipate energy even after hundreds of extension cycles, making them an excellent choice for developing strong and tough materials. Many researchers have tried to mimic biomaterials to enable recoverable energy-dissipating mechanisms. The introduction of dynamic noncovalent bonds-such as hydrogen bonding,^34–36^ metal–ligand,^37–39^ and ionic interactions,^40,41^ as sacrificial bonds into polymer materials has been highly pursued. In these materials, the sacrificial bonds sustain a load under small deformation but preferentially rupture while the structural integrity is preserved, thus dissipating energy and conferring improved mechanical performance. For example, inspired by the modular domain structures and reversible unfolding process in the skeletal muscle protein titin, Guan *et al.* successfully incorporated the quadruple hydrogen-bonding 2ureido-4[1*H*]-pyrimidone (UPy) motif as a reversibly modular cross-linker into a linear synthetic polymer, which exhibited impressive tensile strength and extensibility.^42^ As another type of dynamic bond, metal coordination bonds can also act as sacrificial bonds and have been introduced into polymers to fabricate a regular network structure by coordinating organic ligands and metal ions. Recently, highly stretchable and self-healing poly(dimethylsiloxane) crosslinked by coordination complexes involving multiple metal–ligand bonds were developed (with a strain up to 10000%).^4^ Besides, Xia *et al.* designed an Fe^3+^ cross-linked moldable polymer that exhibited remarkable mechanical properties: a strength of 12.6 MPa, and a strain of 1000%.^43^ However, these polymers still have unsatisfactory stiffness and toughness because the sacrificial bonds in elastomers rely on either metal–ligand interactions or hydrogen bonds. Taken together, it was desirable to develop a new synthesis strategy of polymers with two types of sacrificial bonds, which would enable enhanced mechanical properties and multifunctional behaviors compared to systems with a single type of sacrificial bond.

**Fig. 1:**
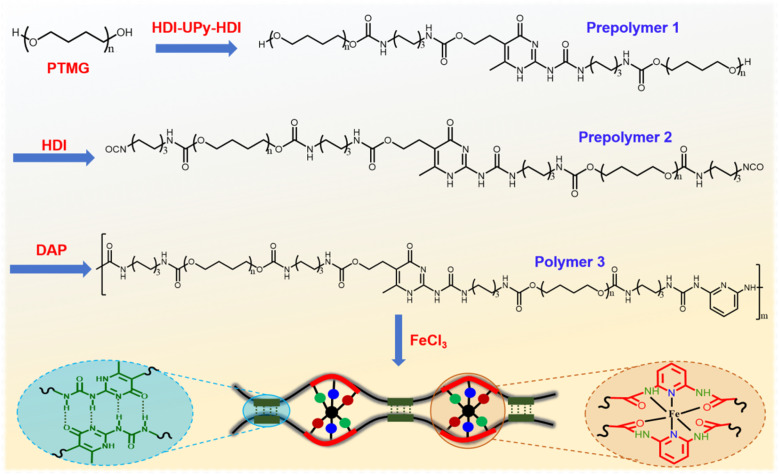
Schematic diagram of the synthesis route of polyurethane elastomer.

In this study, inspired by the biological tissues and mussel byssus, we demonstrated a bioinspired design of polyurethane elastomer, wherein multiple hydrogen bonds (UPy) and metal coordination bonds (DAP-Fe(iii)) were introduced into polymeric networks to improve mechanical toughness. The schematic molecular design was shown in Fig. 1. The self-complementary quadruple hydrogen bonds interactions between UPy dimers were incorporated as the physical cross-linkages, which greatly enhanced the mechanical strength and high stretchability. In addition, strong Fe-coordination bonds could readily break and re-form, which was favourable for energy dissipation during stretching, leading to significantly improved robustness while maintaining stretchability. Owing to accurate design, the synthesized polyurethane elastomer exhibited all the desired properties, mainly including a high tensile stress of ∼30 MPa, a high stretchability of ∼4100%, exceptional toughness of ∼470 MJ m^−3^, excellent self-recoverability and self-healing ability. Owing to the combination of excellent stiffness, toughness and recoverability, it was believed that this synthesis polyurethane elastomers were expected to have enormous potential for application in fabrication of smart material.

## 2 Experimental section

### 2.1 Materials

Polytetramethylene glycol (PTMG, Mn = 2000 g mol^−1^), triethylamine (Et_3_N), 2-acetylbutyrolactone, guanidine carbonate, 2,6-diaminopyridine (DAP) and Iron(iii) chloride anhydrous were purchased from Sigma-Aldrich (Shanghai China). 1,6-Hexyldiisocyanate (HDI, 99%) and dibutyltindilaurate (DBTDL) were obtained from J&K Scientific Ltd. 2,6-Diaminopyridine (DAP) was recrystallized from toluene. PTMG-2000 sample was dried in vacuum at 120 °C for three hours prior to use. Dimethylacetamide (DMAc) was dried over CaH_2_ prior to distillation under reduced pressure. All the other reagents were received from Tianjin Concord Pharmaceutical Chemical Co. Ltd, and used as received without further purification.

### 2.2 Synthesis of polyurethane elastomers

As shown in Fig. S1, the UPy-containing monomer (HDI-UPy-HDI) were synthesized as the procedure reported in the previous study.^44^ Polyurethane elastomer was synthesized according to the route shown in Fig. 1. PTMG-2000 (8.0 g, 4.0 mmol) firstly reacted with HDI-UPy-HDI (1.010 g, 2 mmol) in anhydrous *N*,*N*-dimethylacetmide (DMAc, 15 ml) at 70 °C under nitrogen atmosphere for 1 h, yielding a prepolymer 1. Subsequently, HDI (0.672 g, 4 mmol) was slowly injected into the above reaction solution, and then the reaction was conducted at 70 °C for another 0.5 h, yielding a prepolymer 2. After that, DAP (0.216 g, 2 mmol) and the catalyst dibutyltin dilaurate (DBTDL, 0.5 mol% for isocyanate or alcohol units) in anhydrous *N*,*N*-dimethylacetmide (DMAc, 10 ml) were added into the mixture and further stirred for another 12 h at 70 °C, yielding a polymer 3. Finally, in a ratio of *n* (DAP) : *n* (Fe^3+^) = 2 : 1, anhydrous FeCl_3_ (0.162 g, 1 mmol) in *N*,*N*-dimethylacetamide (DMAc, 5 ml) was added to the above solution to form cross-linked polyurethane. The mixture was stirred for 5 min at 70 °C to provide a uniform network. The solution was poured into a Teflon casting dish dried under 70 °C overnight followed by 70 °C for 48 h under vacuum. After drying, the resulting polymer sheet was peeled off from the Teflon mold to obtain polyurethane elastomer.

### 2.3 Characterization of polyurethane elastomers

#### 2.3.1 Nuclear magnetic resonance (NMR)

Solution NMR experiments were performed on a Bruker AVANCE III NMR spectrometer with a proton resonance frequency of 400.13 MHz. The samples were dissolved in deuterated chloroform, DMSO-*d*_6_ or DMF-*d*_7_ with a small amount of TMS as the internal reference standard.

#### 2.3.2 Fourier transform infrared spectroscopy

The infrared spectra were recorded with a resolution of 8 cm^−1^ and 16 scans per sample, using a Bio-Rad FTS6000 spectrometer and UMA600 microscope equipped with a Linkam FTIR600 heat stage.

#### 2.3.3 Gel permeation chromatography (GPC)

Molecular weight and PDI of polymers were determined by GPC equipped with Hitachi L-2130 HPLC pump, Hitachi L-2350 column oven operated at 25 °C. THF was used as eluents at a flow rate of 1.0 ml min^−1^.

#### 2.3.4 Ultraviolet-visible (UV-vis) spectra

UV-vis absorption recorded on the UV-vis spectrophotometer (Agilent CARY 60). The samples were dissolved in tetrahydrofuran (THF) with a concentration of 0.25 mg ml^−1^.

#### 2.3.5 Scanning electron microscopy (SEM)

SEM was performed by ZEISS Merlin field emission microscopy working at 10 kV voltages.

#### 2.3.6 Mechanical testing

Stress–strain curves were measured on an UTM6103 mechanical testing instrument (Shenzhen Suns Technology Stock Co., Ltd, China) in tensile mode under a strain rate of 100 mm min^−1^ at room temperature. Dog-bone-shaped test strips were prepared from the PU films using a gauge dimension of: 50 mm × 4 mm and thickness of 1.0–1.2 mm measured for each sample with SFJ digital thickness tester.

#### 2.3.7 Differential scanning calorimetry (DSC)

DSC measurements were performed on a Mettler-Toledo DSC1 differential scanning calorimeter with a heating rate of 10 °C min^−1^ under nitrogen atmosphere. About 10 mg samples were encapsulated in 40 µL aluminum pans before measurements.

#### 2.3.8 X-ray diffraction

XRD measurement was carried out on a Bruker Model D8 FOCUS X-ray diffractometer with Cu Kα irradiation (*λ* = 1.5406 A) at a generator voltage of 40 kV and a current of 40 mA.

#### 2.3.9 Small angle X-ray scattering (SAXS) measurement

The SAXS experiments were performed at room temperature using a Bruker Nanostar SAXS system. The long period *d* was inversely related to the wave vector at the scattering peak, *d* = 2π/*q*.

### 2.4 Atomic force microscope (AFM) measurement

Samples for AFM imaging were prepared by solution casting relatively thin films from 1% DMAc solutions on freshly cleaved mica plates. Excess was wicked away with filter paper. The AFM imaging was performed in air at room temperature through a Multimode 8 system (Bruker AXS, Santa Barbara, USA) operated in tapping mode.

#### 2.4.1 Thermogravimetric analysis (TGA)

TGA experiments were conducted on a NETZSCH TG 209 instrument at a linear heating rate of 10 °C min^−1^ from 25 to 600 °C under nitrogen atmosphere.

#### 2.4.2 Dynamic mechanical analysis (DMA)

DMA measurements were performed on a TA Instruments Q800 in tension mode. Samples were carried out in the temperature range of −80–140 °C under a nitrogen atmosphere. The frequency was 1 Hz and the heating rate was 5 °C min^−1^.

## 3 Results and discussion

### 3.1 Design, synthesis and characterization of polyurethane elastomer

Several samples of PU-UPy(*x*)-DAP were prepared in a step-growth manner, where *x* represents the content of UPy motifs. The PU-UPy(*x*)-DAP polymers were used as binding motifs to coordinate Fe(iii) ions, which further act as cross-linkers to construct robust polymer networks (PU-UPy(*x*)-DAP-Fe). In order to understand the role of multiple hydrogen bonds (UPy) and metal coordination bonds, six PU samples with a fixed content of DAP chain extenders were prepared as listed in Table 1, where the molar ratio between HDI-UPy-HDI and HDI was varied accordingly. For example, PU-UPy1-DAP-Fe indicated that the molar ratio between UPy motifs and DAP was 1 : 1, corresponding to a molar ratio of 2 : 1 between DAP and Fe(iii) ions. Details about the raw materials used for sample preparation were summarized in Table 1.

**Table 1:** Raw materials used for the preparation of polymer samples and their molecular weight

Sample	PTMG (mmol)	HDI-UPy-HDI (mmol)	HDI (mmol)	DAP (mmol)	FeCl_3_ (mmol)	Mn^a^ (g mmol^−1^)	Mw^a^ (g mmol^−1^)	PDI
PU-UPy0-DAP	4.0	0	6.0	2.0	0	53890	73063	1.35
PU-UPy0-DAP-Fe	4.0	0	6.0	2.0	1.0	—	—	—
PU-UPy0.5-DAP	4.0	1.0	5.0	2.0	0	48980	83640	1.70
PU-UPy0.5-DAP-Fe	4.0	1.0	5.0	2.0	1.0	—	—	—
PU-UPy1-DAP	4.0	2.0	4.0	2.0	0	51408	86353	1.68
PU-UPy1-DAP-Fe	4.0	2.0	4.0	2.0	1.0	—	—	—

a Determined by THF-GPC using polystyrene standards.

**Fig. 2:**
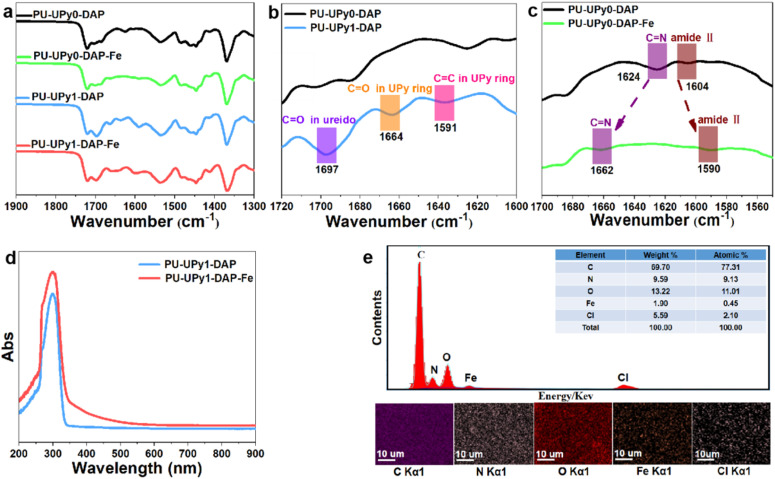
(a–c) FTIR spectra of different samples. (d) UV-vis spectra of PU-UPy1-DAP (blue), and PU-UPy1-DAP-Fe(red). (e) SEM-EDS analysis and mapping images of PU-UPy1-DAP-Fe.

^1^H NMR demonstrated the successful preparation of bulk PTMG-UPy-DAP, as indicated by the presence of characteristic peaks of UPy and DAP segments in polymeric backbones (Fig. S4), suggesting the successful incorporation of UPy and DAP functionality in the prepared polymers. In addition, Fourier transform infrared (FTIR) spectroscopy were used for characterizing the structures of the elastomer. As shown in Fig. 2a and b (full spectra provided in the SI as Fig. S5), PU-UPy1-DAP sample demonstrated the typical UPy characteristic peak at around 1697, 1664 and 1591 cm^−1^. Specifically, the peaks at 1697 cm^−1^, 1664 cm^−1^ and 1591 cm^−1^ could be ascribed to the signals of C=O stretching in ureido, C=O stretching and C=C stretching in UPy rings, respectively.^35,44^ This indicated that UPy unit was successfully synthesized in the polymer backbone. In addition, from Fig. 2c, a characteristic absorption peak at 1624 cm^−1^ was observed on a spectrum of DAP ligand, which was assigned to the C=N vibration modes of free pyridine.^4^ Upon complexation with Fe^3+^, the signal of C=N vibration shifted from 1624 to 1662 cm^−1^, indicating the coordination of pyridine with Fe^3+^. Besides, amide band II at 1604 cm^−1^ of DAP shifted to 1590 cm^−1^ after complexation with Fe^3+^, indicating that the nitrogen of the amide group was coordinated with Fe^3+^.^43^ Furthermore, the coordination of DAP ligand in polymer with ferric chloride (FeCl_3_) was investigated by UV-vis titration. As shown in Fig. 2d, compared wih PU-UPy1-DAP, a long wavelength tail between 350 nm and 550 nm of PU-UPy1-DAP-Fe should be attributed to the Fe–N/O (DAP groups) charge transfer band.^4^ Moreover, the elemental mapping analysis of energy-dispersive spectroscopy (EDS) reveals that Fe^3+^ ions were successfully incorporated and uniformly dispersed in the supramolecular networks (Fig. 2e).

### 3.2 Investigation of mechanical properties of polyurethane elastomer

**Fig. 3:**
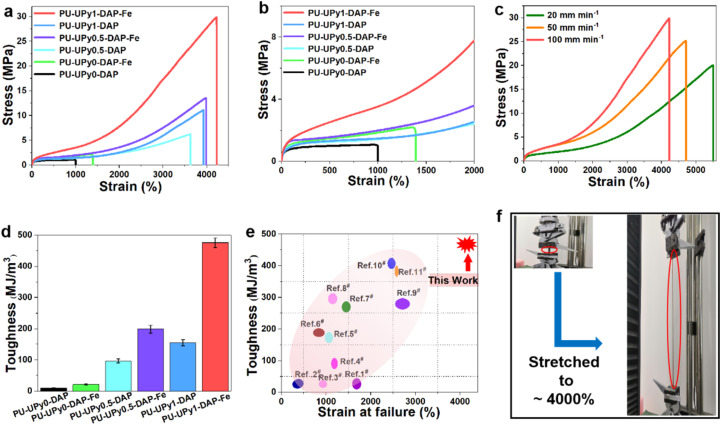
(a) Stress–strain curves of PU elastomers obtained from tensile test at a stretching rate of 100 mm min^−1^. (b) Partial enlargement of Fig. 3a for clarity. (c) Stress–strain curve of the PU-UPy1-DAP-Fe film at different speeds. (d) Comparison of toughness of different samples. The toughness was calculated from the area under stress–strain curve up to the strain at break. (e) Comparison of the PU-UPy1-DAP-Fe film with previously reported highly stretchable PU elastomers. (f) Photographs of PU-UPy1-DAP-Fe strip (marked by red ellipse) before and after being stretched.

The mechanical properties of elastomer were characterized *via* static uniaxial tensile testing at room temperature, as shown in Fig. 3a and b, besides the detailed mechanical parameters were presented in Table S1 in SI. The mechanical property tests illustrated that these samples were classical elastomers because they did not exhibit yielding phenomena during elongation. In addition, their properties were varied by adjusting the quadruple hydrogen bonds (UPy) and coordination bonds (DAP-Fe). With the amount of UPy molar ratios increasing from 0 to 1, the mechanical properties of the elastomers were enhanced obviously, indicating a higher cross-linking density and a stronger hydrogen-bonded network. Indeed, it taken ∼11 kcal mol^−1^ to fully unfold the UPy folded module, thus, during stretching, the continuous unfolding and disassociation of UPy dimers enables the polymers with high extensibility and remarkable toughness. Moreover, Fe(iii) could form strong coordination bonds with DAP binding motifs and the bonding energies of Fe(iii)-Npyridyl, Fe(iii)-Namido and Fe(iii)-Oamido bonds were further estimated as 145.0, 82.7 and 40.7 kcal mol^−1^, respectively. The stronger Fe(iii)-Npyridyl bond was comparable to typical covalent bonds, which maintained the integrity of the network and conferring corresponding stiffness and elasticity, whereas the weaker Fe(iii)-Oamido bond was almost as weak as hydrogen bonding which dissipate strain energy by efficient reversible bond rupture and reforming. Therefore, a proper combination of strong bonds and weak bonds in a single polymeric network was critical for increasing the mechanical strength but without sacrificing the maximum elongation and toughness. Fig. 3c exhibited that the stretchability of PU-UPy1-DAP-Fe film was strongly dependent on the stretching speed. A maximum fracture strain up to 5500 ± 187% could be achieved for a sample at a loading rate of 20 mm min^−1^. Because of the decreased strain speed, there was more time available for broken dynamic bonds to reform, significantly increasing the fracture tolerance. Compared with that of PU-UPy0-DAP elastomer, the toughness of PU-UPy1-DAP-Fe elastomer which was driven by the synergistic effect between quadruple hydrogen bonds and coordination bonds were increased by about 51 times. In addition, compared with previously reported elastomers, PU-UPy1-DAP-Fe elastomer has high toughness of ∼470 MJ m^−3^ and high stretchability of ∼4100% (Fig. 3e).

**Fig. 4:**
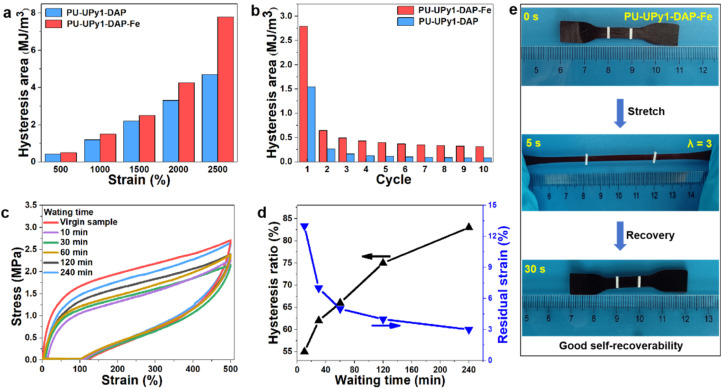
(a) Dissipated energy during loading–unloading processes under different strains. (b) Dissipated energy during ten successive loading–unloading processes with a strain of 1500% in every cycle. (c) Cyclic tensile test curves of PU-UPy1-DAP-Fe loaded to a strain of 500% with delay time ranging from 0 to 240 min. (d) The hysteresis ratio and residual strain of PU-UPy1-DAP-Fe sample loaded to a strain of 500% with delay time ranging from 0 to 240 min. (e) Optical images for PU-UPy1-DAP-Fe film that could quickly revert to its original length after being stretched to 300% strain.

Cyclic tensile tests were conducted to investigate the energy dissipation caused by the dynamic nature of the quadruple hydrogen bonds and coordination bonds interactions. PU-UPy1-DAP and PU-UPy1-DAP-Fe displayed significant hysteresis loops (SI, Fig. S6). The hysteresis energy (area surrounded by tensile–recovery curves) increased gradually as the strain increased and then began to grow rapidly at higher strains (Fig. 4a). During ten successive loading-unloading cycles, the hysteresis area of PU-UPy1-DAP-Fe was larger than PU-UPy1-DAP but decreased considerably after the first stretching cycle (Fig. 4b). These results confirmed that force-induced rupture of hierarchical hydrogen-bonding and coordination bonds serving as sacrificial bonds contributed to effective energy dissipation. In addition, the energy dissipation of PU-UPy1-DAP-Fe was more than PU-UPy1-DAP and the gap was getting bigger as the strain increased, demonstrating that the polymers with two types of sacrificial bonds have a better energy dissipation mechanism compared to the system with single sacrificial bonds. To elucidate the recovery properties of elastomer, PU-UPy1-DAP-Fe was repeatedly loaded to a 500% strain and unloaded with different waiting time ranging from 0 to 240 min, as shown in Fig. 4c and d. Large hysteresis and notable residual strain were observed in this first loading-unloading cycle. The second circle with a delay time of 10 min shown a smallest hysteresis loop, due to the dissociation of non-covalent bond interactions during the first loading cycle. As the delay time increased, the surviving non-covalent bond interactions could re-associate with each other gradually, thus accelerating the recovery process, contributing to the increment of hysteresis area and recovery of the loading curves. The recovery ratio increased after waiting for long time, and finally reached up to 83% of the original value after a delay of 240 min. The residual strain was also eliminated with time. In short, all the results have demonstrated the efficient energy dissipation induced by quadruple hydrogen bonds and coordination bonds interactions, which endow the elastomer with repeatable deformation recovery (good deformation recovery from an elongation of 300%, Fig. 4e).

### 3.3 Investigation of microstructure of polyurethane elastomer

**Fig. 5:**
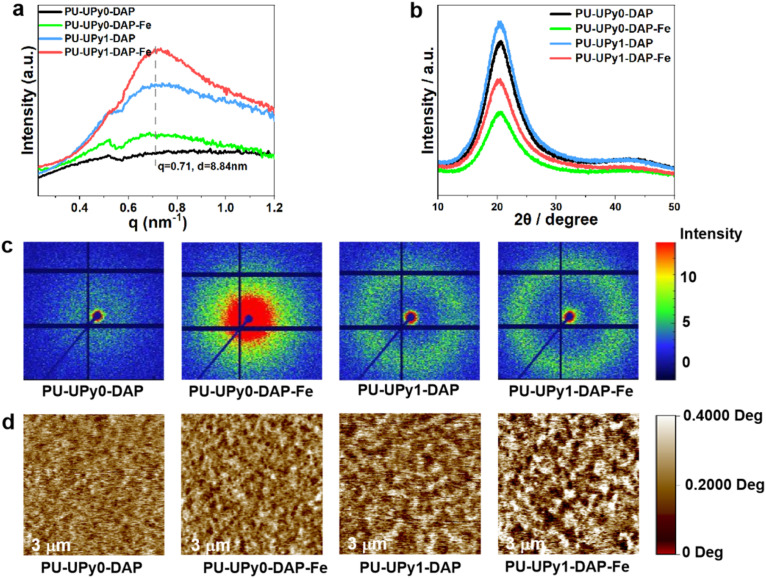
(a) SAXS scatterning patterns of PU samples. (b) XRD profiles of PU samples. (c) 2D SAXS patterns of PU samples. (d) AFM images of PU samples.

A universal characteristic of segmented polyurethane was the microphase separation due to the incompatibility between the hard and soft domains. Herein, the microphase separation of the PU elastomers was studied by small-angle X-ray scattering (SAXS) and atomic force microscope (AFM). As shown in the and *Iq*^2^ − *q* curve (Fig. 5a) and 2D-SAXS graph (Fig. 5c), the PU-UPy0-DAP sample without quadruple hydrogen bonds and coordination bonds exhibited an unobvious short-range ordered structure. Interestingly, the SAXS patterns of the PU-UPy0-DAP-Fe elastomer exhibited a clear principal scattering peak at around *q* ∼0.71 nm^−1^, indicating the existence of phase separated domains in the elastomers, which was also confirmed by 2D-SAXS scattering patterns. This was because the DAP groups coordinated with Fe^3+^ ions were isotropically dispersed in the dynamic hard domains, thus promoting phase separation. Moreover, in PU-UPy1-DAP and PU-UPy1-DAP-Fe samples which added the content of UPy motifs, the peak at *q* ∼0.71 nm^−1^ became more obvious and stronger. Besides, a broad or narrow circular scattering halo in 2D-SAXS scattering patterns, which was the typical feature of microphase separation between soft and hard domains, could be seen in the samples with UPy motifs. In fact, ureidopyrimidinone (UPy) featured a donor-donor-acceptor-acceptor (DDAA) hydrogen-bonding motif, which enabled it to self-assemble into stable dimers.^35,42^ This confered stronger intermolecular interactions on the hard segments compared with conventional urea/urethane groups, thereby significantly enhancing the cohesion of the hard segments. Meanwhile, the strong intermolecular interactions of UPy dimers driven the aggregation of hard segments, facilitating the formation of more ordered and larger-sized hard-phase microdomains, which consequently improved the microphase separation of the PU elastomer. Furthermore, atomic force microscope was used to detect the detailed morphology of the microdomain structure in PU elastomers. Worm-like hard domains (bright) and soft domains (dark) could be clearly observed in AFM, as shown in Fig. 5d. Indeed, the PU-UPy1-DAP-Fe with quadruple hydrogen bonds and coordination bonds embedded in hard segments exhibited the most distinctive microphase separation with a periodic length around 8.84 nm (*D* = 2π/*q*), which possibly gave rise to the exceptional stretchability and super toughness of the samples. Since the crystallization of the hard domains would seriously affect the mechanical properties, we carried out the XRD experiments for specimens. The XRD patterns of the samples before stretching were shown in Fig. 5b. All one dimensional curves feature one broad peak around 21°, suggesting the presence of amorphous state without obvious crystal structure.

### 3.4 Thermomechanical behaviors of PU networks

**Fig. 6:**
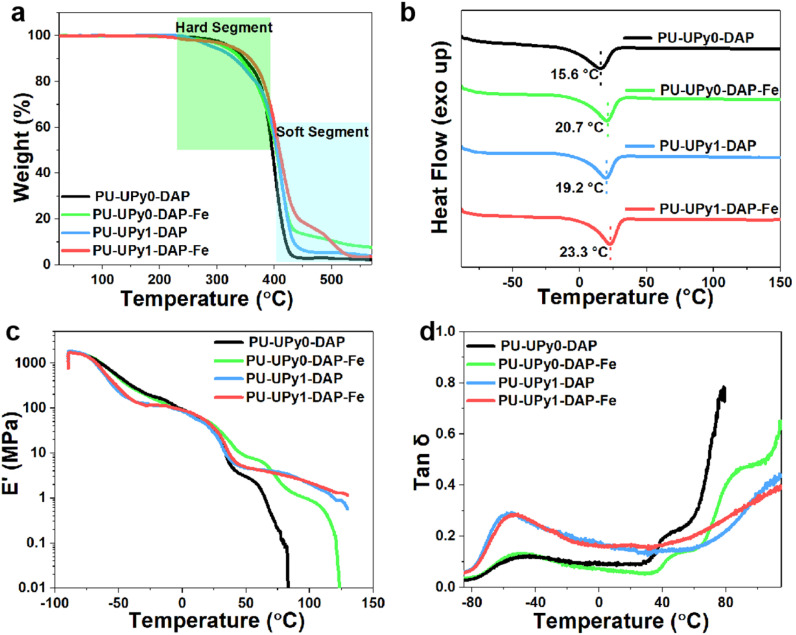
(a) TGA curves of the elastomers. (b) DSC curves of the elastomers. (c) and (d) Storage modulus (*E*′) and tan *δ* curves as a function of temperature obtained from the DMA measurements.

The thermal stability of PU samples was analyzed by thermos gravimetric analysis. As shown in Fig. 6a, all samples exhibit two distinct weight loss steps at 250–370 °C and 370–430 °C. The first stage was attributed to decomposition of hard domains of the PU. The second stage was related to the decomposition of the soft domains. Besides, it was found that quadruple hydrogen bonds and metal coordination bonds endow the materials a higher temperature resistance, leading degradation temperatures shift slightly toward higher temperatures (Fig. 6b). The broad endothermic transition peak which was assigned to the small size imperfect crystals formed in the soft segment had shift from 15.6 to 23.3 °C, indicating physical crosslinking hinders the movement of polymer chains, thus leading to a higher melting temperature. To further illustrate the chain mobility and viscoelastic behavior of the elastomers, the dynamic mechanical analysis (DMA) were tested, as shown in Fig. 6c and d. Compared with samples which have UPy motifs, the PU samples without UPy motifs were softened and could not maintain its shape when the temperatures higher than 50 °C. In the tan *δ* curves (Fig. 6d), the first peak of all samples indicated the glass transition of PTMG. The second peak of samples (PU-UPy0-DAP and PU-UPy0-DAP-Fe) corresponded to the melting of the crystalline PTMG domains. However, the peak for the melting of PTMG crystallites was not obvious for the PUs containing UPy motifs (PU-UPy1-DAP and PU-UPy1-DAP-Fe), which maybe overlapped with the third broad peak begin at 50 °C corresponding to the dissociation of high-density hydrogen bonds (UPy) and metal coordination bonds, demonstrating that non-covalent bond interactions may impose restrictions on polymer dynamics.

### 3.5 Self-healing and recycling properties of polyurethane elastomer

**Fig. 7:**
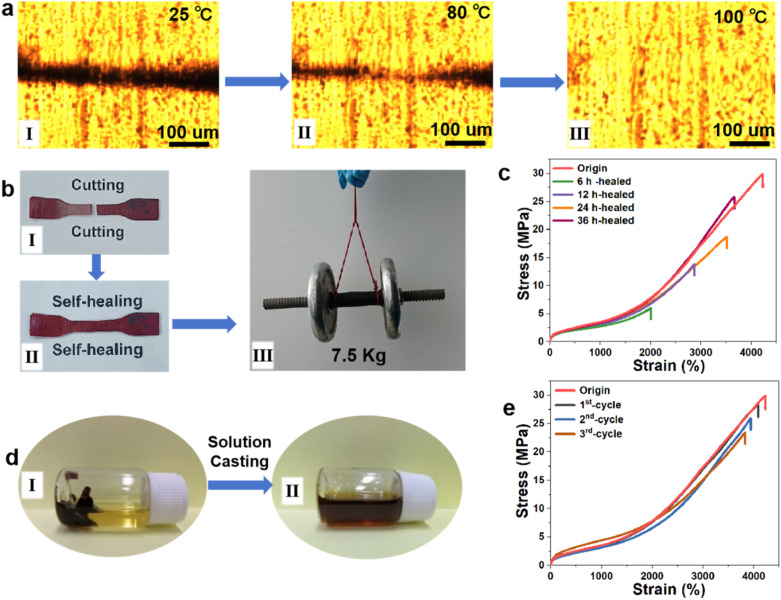
(a) Optical microscopy images of artificially scratched PU-UPy1-DAP-Fe film and healing process. All the samples were kept at each temperature for 5 min and then photos were taken. (b) Digital photographs showing the healing of the fractured elastomer (I,II). The healed elastomer can lift five weights with a total weight of 7.5 kg (III). (c) Typical stress–strain curves of the intact and healed elastomer after 6 h, 12 h, 24 h, and 36 h, respectively. (d) Demonstration of the recyclability of elastomer by solution casting. (e) Stress–strain curves of the intact and recycled elastomer after the first, second, and third cycles.

The use of thermal reversible bonds (quadruple hydrogen bonds and metal coordination bonds) as the cross-linkages has rendered the PU-UPy1-DAP-Fe elastomer healable and recyclable. As shown in Fig. 7a, optical microscopy was adapted to observe the healing of scratches on the sample surface. The scratched surface on the elastomer almost vanish within 5 min at 100 °C, showing excellent self-healing ability. As discussed in Fig. 6c and d above, when the temperature reaches 100 °C, most quadruple hydrogen bonds and metal coordination bonds within the PU network structure undergo sufficient dissociation. This intensified the mobility of polyurethane segments, facilitated the diffusion and fusion of the damaged interface, and ultimately achieved excellent self-healing performance. To quantitatively evaluate self-healing ability, a dumbbell PU-UPy1-DAP-Fe elastomer (4.0 mm in width, 1 mm in thickness) was cut into two separate pieces, and brought into contact and healed at 100 °C for different time. The reconnected elastomer after 36 h of repair could hold a weight of up to 7.5 kg (Fig. 7b), which indicated that the reversible bonds greatly facilitated and accelerated the healing of polymer networks. As shown in Fig. 7c, with the prolonging of healing time, the healing efficiency of PU-UPy1-DAP-Fe elastomer were significantly enhanced due to sufficient hydrogen bond exchanges, metal coordination bonds reforms and polymer chain diffusions. After heating for 36 h, it was found out that the strain at break and stress at break of the elastomer have recovered by about 86% and 83%, respectively, which well indicated the good self-healing ability of the elastomer. Despite the decrease in stress at break and tensile extensibility for the self-healed PU-UPy1-DAP-Fe sample, it could still be recycled by using traditional processing methods such as solution casting (Fig. 7d). The PU-UPy1-DAP-Fe elastomer was cut into millimeter-sized fragments and then dissolved in DMAc to obtain a homogeneous solution. After solvent evaporation, a new defect-free PU-UPy1-DAP-Fe elastomer was obtained. As shown in Fig. 7e, the stress–strain curves of the elastomers recycled in the first cycle almost the same as the pristine one. In addition, the recycling ability in terms of toughness could still reach 75% after three cycles, demonstrating their excellent recycling and reprocessing ability.

## 4 Conclusion

In summary, inspired by the biological tissues and mussel byssus, we have developed a tough and strong elastomer, wherein the multiple hydrogen bonds (UPy) and metal coordination bonds (DAP-Fe(iii)) were introduced into polymeric networks to tune the mechanical properties and self-healing ability of the elastomer. The self-complementary quadruple hydrogen bonding interactions between UPy dimers were incorporated as the physical cross-linkages, which greatly enhanced the mechanical strength and high stretchability. In addition, the strong Fe-coordination bonds could readily break and re-form, which was favourable for energy dissipation on stretching, leading to the significantly improved robustness while maintaining stretchability. Owing to the reasonable design, the synthesized elastomer exhibited all the desired properties, mainly including a high tensile stress of ∼30 MPa, exceptional toughness of ∼470 MJ m^−3^, a high stretchability of ∼4100%, excellent self-recoverability and self-healing ability. We believe that this bioinspired strategy with multiple hydrogen bond and metal coordination bonds offer an efficient yet facile way to improve self-healing and mechanical properties of many other advanced elastomers, which facilitates to expand the scopes of applications.

## Author contributions

Li Jian: writing – original draft, methodology, conceptualization, funding acquisition. Fubo Ma: writing – review & editing, formal analysis. Jintao Ji: writing – review & editing, supervision. Yuanzhi Qu: formal analysis. Xiaoxiao Ni: investigation.

## Conflicts of interest

We declare that there were no financial conflicts of interest in the experimental design, data analysis, and paper writing process of this study. In addition, there is no personal relationship that affects the rigor of the experiment, the objectivity, and authenticity of the conclusions.

## Supplementary Material

RA-016-D5RA08303F-s001

## Data Availability

Direct request to the corresponding author. Supplementary information (SI): supplementary data associated with this article can be found in the online version at RSC Advances. See DOI: https://doi.org/10.1039/d5ra08303f.
