# Supplementary material for: Bioinspired super-tough polyurethane elastomers with block modules using sacrificial bonds
Source: RSC Adv. 2026 Jan 16;16(4):3780–90. doi: 10.1039/d5ra08303f (PMC12810322; doi:10.1039/d5ra08303f)
Supplement: RA-016-D5RA08303F-s001 [file RA-016-D5RA08303F-s001.pdf]

Supplementary Information (SI) for RSC Advances.
This journal is © The Royal Society of Chemistry 2026

SUPPORTING INFORMATION

Bioinspired Super-tough Elastomers with Block Modules using Sacrifical Bonds

Jian Li[1,2*], Fubo Ma[2], Jintao Ji[3], Yuanzhi Qu[4], Xiaoxiao Ni[4]

[1]State Key Laboratory of Deep Oil and Gas, China University of Petroleum (East China), Qingdao 266580, China

[2]School of Petroleum Engineering, China University of Petroleum (East China), Qingdao 266580, China;

[3]Hebei Drilling and Production Equipment Manufacturing Branch, PetroChina Company Limited, Renqiu 062552, China;

[4]Drilling Fluid Research Institute, CNPC Engineering Technology R&D Company Limited, Beijing 102206, China

*Corresponding author: cuplijian@sina.com

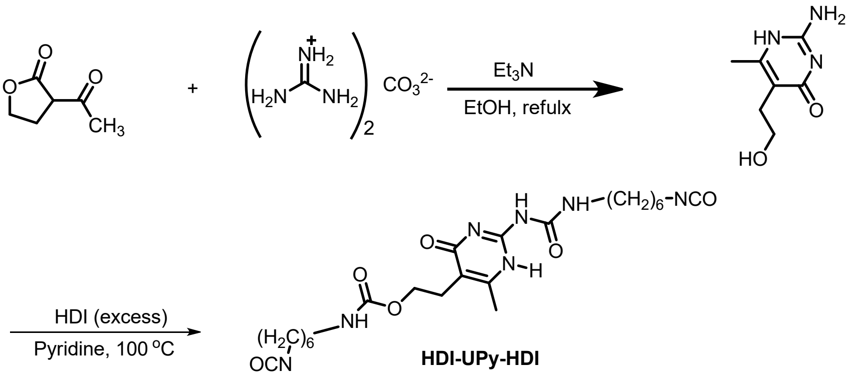

FIGURE S1. Schematic synthesis pathway of HDI-UPy-HDI.

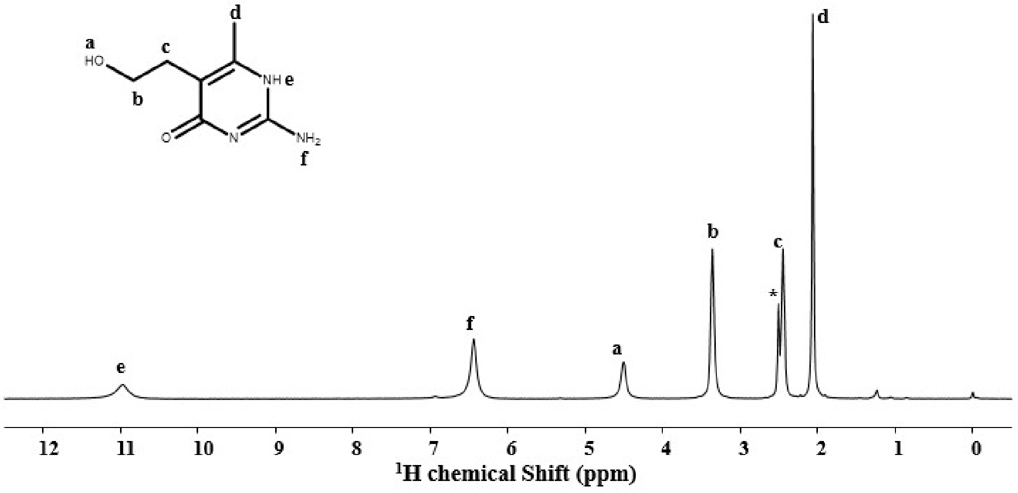

FIGURE S2. ^{1}H NMR spectrum of UPy precusor in DMSO-d6. “*” denotes the proton signals of DMSO-d6.

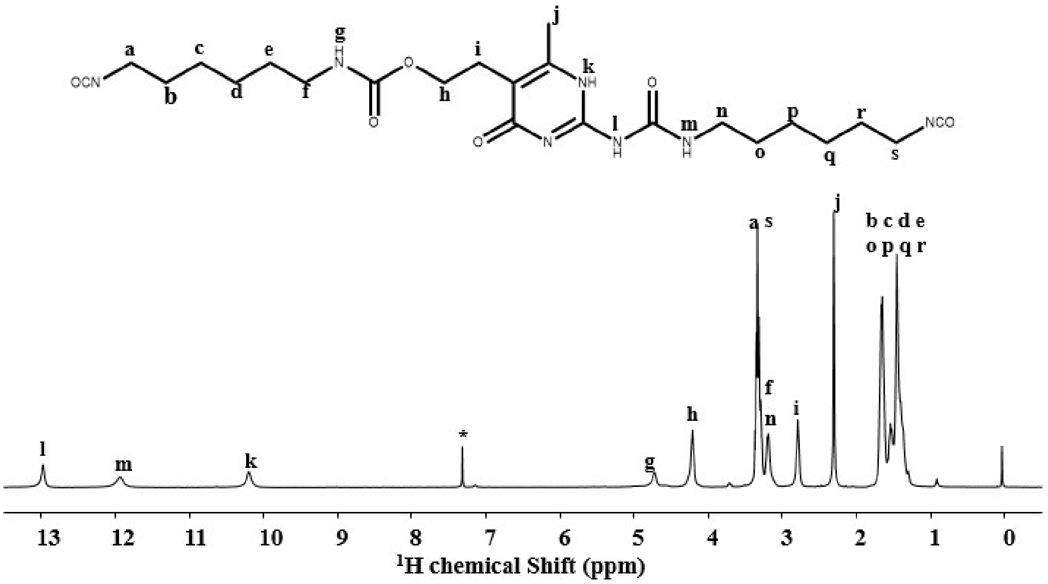

FIGURE S3. ^{1}H NMR spectrum of HDI-UPy-HDI in $CDCl_3$, “*” denotes the solvent signal.

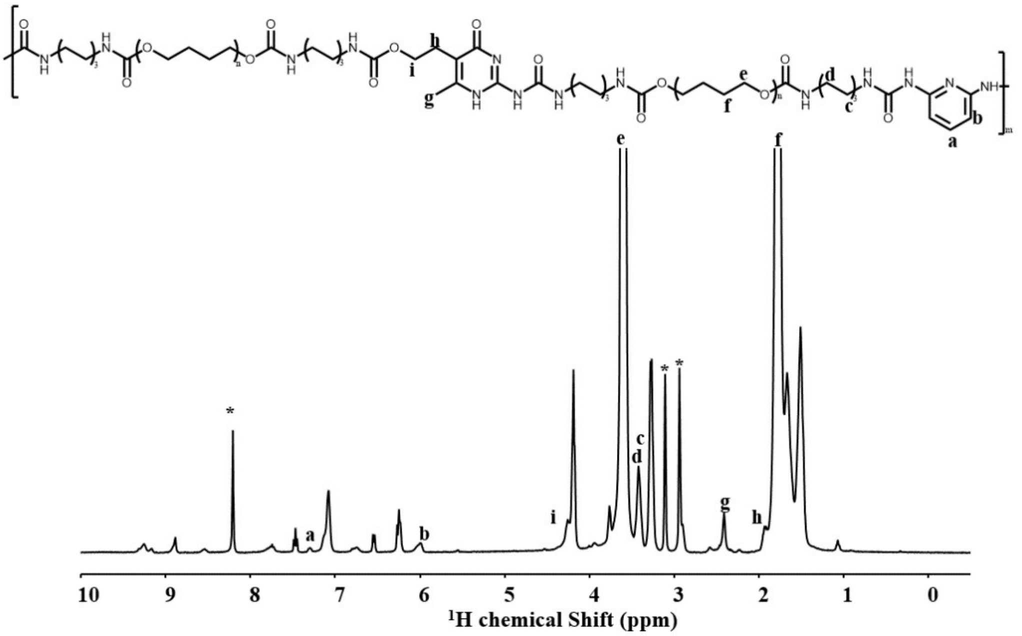

FIGURE S4. ^{1}H NMR spectrum of PU-UPy1-DAP in DMF-d7, “*” denotes the solvent signal. ^{1}H (400 MHz, DMF-d7, δ ppm).

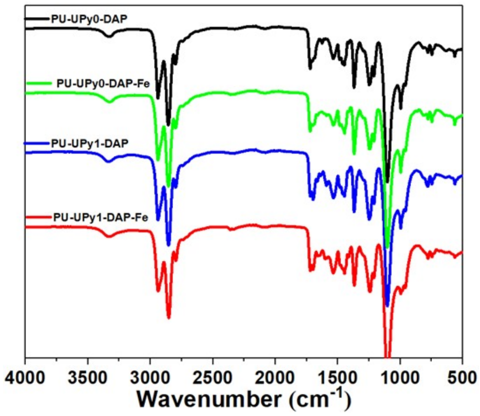

FIGURE S5. FTIR spectra of different samples.

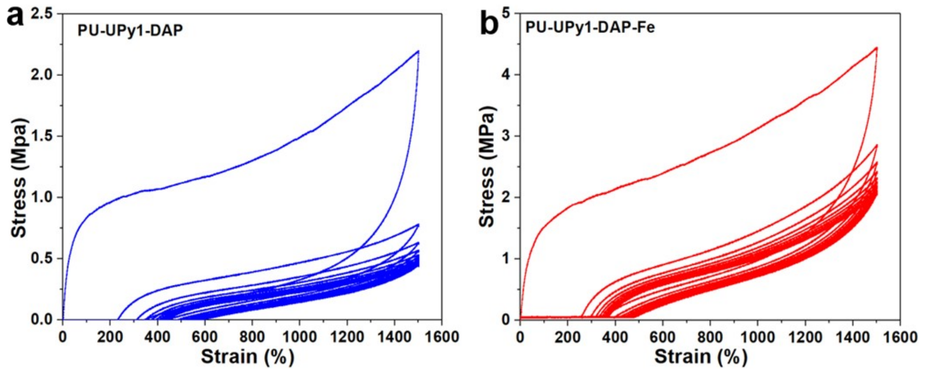

FIGURE S6. Ten successive loading-unloading cycles with 1500% strain of PU-UPy1-DAP(a) and PU-UPy1-DAP-Fe(b) elastomers.

Table S1. The detailed mechanical parameters for the Preparation of Polymer Samples

**Sample**	**Tensile strength (MPa)**	**Young's Modulus (Mpa)**	**Elongation at break (%)**	**Toughness (MJ/m^3)**
PU-UPy0-DAP	1.0 ± 0.5	3.24 ± 0.4	986 ± 87	9.62 ± 1.23
PU-UPy0-DAP-Fe	2.0 ± 0.5	5.25 ± 0.5	1368 ± 89	22.25 ± 2.12
PU-UPy0.5-DAP	6.2 ± 0.6	4.79 ± 0.4	3626 ± 123	97.08 ± 6.77
PU-UPy0.5-DAP-Fe	13.5 ± 1.5	7.37 ± 0.3	3966 ± 108	199.18 ± 12.53
PU-UPy1-DAP	11.5 ± 1.8	4.59 ± 0.3	3921 ± 158	155.25 ± 18.91
PU-UPy1-DAP-Fe	30.2 ± 2.6	8.34 ± 0.3	4239 ± 152	475.94 ± 24.08

Table S2. A summary for mechanical properties of recently reported synthetic material

**Sample**	**Toughness (MJ/m^3)**	**Elongation at break (%)**	**Tensile strength (MPa)**	**Source**
PU/Fe(III) Coordination +(UPy)	401	4139	25.9	This work
PU/Hydrogen Bonds and Coordination Bonds	14.7	1700	2.6	Ref.1[1]
PU/ disulfide bond	21.4	308	18.9	Ref.2[2]

PU/Aromatic Disulfide Bonds	26.9	923	6.8	Ref.3[3]
PU/ Cu(II) Coordination	87	1200	14.8	Ref.4[4]
PU/Polydopamine Functionalized Graphene	175	1033	40	Ref.5[5]
PU/Ionic Interactions	189	859	48	Ref.6[6]
PU/Unmodified Lignin	264	1394	33	Ref.7[7]
PU/Cellulose Nanocrystals	290	1110	49	Ref.8[8]
PU/ZrO_2 nanoparticle	280	2714	23.7	Ref.9[9]
PU/Thiol-Michael Adduct Crosslinker +UPy	400	2500	40	Ref.10[10]
PU/Quadruple Hydrogen Bond(UPy)	386	2580	48	Ref.11[11]

Reference

[1] X. Wu, J. Wang, J. Huang, S. Yang, Robust, stretchable, and self-healable supramolecular elastomers synergistically cross-linked by hydrogen bonds and coordination bonds. *ACS Appl. Mater. Interfaces.* ***2019***, *11, 7387-7396.*

[2] W.B. Ying, Z. Yu, D.H. Kim, K.J. Lee, H. Hu, Y. Liu, Z. Kong, K. Wang, J. Shang, R. Zhang, J. Zhu, R.W. Li, Waterproof, highly tough, and fast self-healing polyurethane for durable electronic skin. *ACS Appl. Mater. Interfaces.* ***2020***, *12, 11072-11083.*

[3] S.M. Kim, H. Jeon, S.H. Shin, S.A. Park, J. Jegal, S.Y. Hwang, D.X. Oh, J. Park, Superior toughness and fast self-healing at room temperature engineered by transparent elastomers. *Adv. Mater.* ***2018***, *30, 1705145.*

[4] L. Zhang, Z. Liu, X. Wu, Q. Guan, S. Chen, L. Sun, Y. Guo, S. Wang, J. Song, E.M. Jeffries, C. He, F.L. Qing, X. Bao, Z. You, A highly efficient self-healing elastomer with unprecedented mechanical properties. *Adv. Mater.* ***2019***, *31, 1901402.*

[5] K. Chen, Q. Tian, C. Tian, G. Yan, F. Cao, S. Liang, X. Wang, Mechanical reinforcement in thermoplastic polyurethane nanocomposite incorporated with polydopamine functionalized graphene nanoplatelet. *Ind Eng Chem Res.* ***2017***, *56, 11827-11838.*

[6] H. Daemi, S. Rajabi-Zeleti, H. Sardon, M. Barikani, A. Khademhosseini, H. Baharvand, A robust super-tough biodegradable elastomer engineered by supramolecular ionic interactions. *Biomaterials.* ***2016***, *84, 54-63.*

[7] W. Liu, C. Fang, S. Wang, J. Huang, X. Qiu, High-performance lignin-containing polyurethane elastomers with dynamic covalent polymer networks. *Macromolecules.* ***2019***, *52, 6474-6484.*

[8] A. Pei, J.M. Malho, J. Ruokolainen, Q. Zhou, L.A. Berglund, Strong nanocomposite reinforcement effects in polyurethane elastomer with low volume fraction of cellulose nanocrystals. *Macromolecules.* ***2011***, *44, 4422-4427.*

[9] C. Zhang, L. Xia, P. Lyu, Y. Wang, C. Li, X. Xiao, F. Dai, W. Xu, X. Liu, B. Deng, Is it possible to fabricate a nanocomposite with excellent mechanical property using unmodified inorganic nanoparticles directly? *ACS Appl. Mater. Interfaces.* ***2018***, *10, 15357-15363.*

[10] Z. Yang, F. Wang, C. Zhang, J. Li, R. Zhang, Q. Wu, T.H. Chen, P.C. Sun, Bio-inspired self-healing polyurethanes with multiple stimulus responsiveness. *Polym Chem.* ***2019***, *10, 3362-3370.*

[11] Y. Song, Y. Liu, T. Qi, G.L. Li, Towards dynamic but supertough healable polymers through biomimetic hierarchical hydrogen-bonding interactions. *Angew. Chem., Int. Ed.* ***2018***, *130, 14034-14038.*
